# Gadoxetic Acid-Based MRI for Decision-Making in Hepatocellular Carcinoma Employing Perfusion Criteria Only—A Post Hoc Analysis from the SORAMIC Trial Diagnostic Cohort

**DOI:** 10.3390/curroncol29020051

**Published:** 2022-01-27

**Authors:** Max Seidensticker, Ingo G. Steffen, Irene Bargellini, Thomas Berg, Alberto Benito, Bernhard Gebauer, Roberto Iezzi, Christian Loewe, Musturay Karçaaltincaba, Maciej Pech, Christian Sengel, Otto van Delden, Vincent Vandecaveye, Christoph J. Zech, Jens Ricke

**Affiliations:** 1 Department of Radiology, Ludwig-Maximilians-University Munich, 81377 München, Germany; Ingo.steffen@charite.de (I.G.S.); Jens.ricke@med.lmu.de (J.R.); 2 Department of Interventional Radiology, Pisa University Hospital, 56100 Pisa, Italy; irene.bargellini@ao-pisa.toscana.it; 3 Division of Hepatology, Department of Medicine II, Leipzig University Medical Center, 04103 Leipzig, Germany; Thomas.berg@medizin.uni-leipzig.de; 4 Department of Radiology, Clínica Universidad de Navarra, 31008 Pamplona, Spain; albenitob@unav.es; 5 Department of Radiology, Charité-Universitätsmedizin Berlin, 10117 Berlin, Germany; Bernhard.gebauer@charite.de; 6 Dipartimento di Diagnostica per Immagini, Radioterapia Oncologica ed Ematologia, Fondazione Policlinico Universitario A. Gemelli IRCCS, UOC di Radiologia, 00168 Rome, Italy; roberto.iezzi@policlinicogemelli.it; 7 Section of Cardiovascular and Interventional Radiology, Department of Bioimaging and Image-Guided Therapy, Medical University of Vienna, 1090 Vienna, Austria; christian.loewe@meduniwien.ac.at; 8 Department of Radiology, Hacettepe University School of Medicine, Ankara 06230, Turkey; mkarcaal@hacettepe.edu.tr; 9 Department of Radiology and Nuclear Medicine, University of Magdeburg, 39120 Magdeburg, Germany; Maciej.pech@med.ovgu.de; 10 Radiologie Interventionnelle Vasculaire et Percutanée, CHU de Grenoble, 38700 Grenoble, France; CSengel@chu-grenoble.fr; 11 Department of Radiology and Nuclear Medicine, Academic Medical Center, University of Amsterdam, 1012 WX Amsterdam, The Netherlands; o.m.vandelden@amsterdamumc.nl; 12 Department of Radiology, University Hospitals Leuven, 3000 Leuven, Belgium; vincent.vandecaveye@uzleuven.be; 13 Radiology and Nuclear Medicine, University Hospital Basel, University of Basel, 4031 Basel, Switzerland; Christoph.Zech@usb.ch

**Keywords:** computed tomography, contrast dynamics, gadoxetic acid, hepatocellular carcinoma, magnetic resonance imaging, transitional phase, venous phase, wash-out

## Abstract

The value of gadoxetic acid in the diagnosis of hepatocellular carcinoma (HCC), based on perfusion criteria, is under dispute. This post-hoc analysis of the prospective, phase II, randomized, controlled SORAMIC study compared the accuracy of gadoxetic acid-enhanced dynamic magnetic resonance imaging (MRI) (arterial, portovenous, and venous phase only) versus contrast-enhanced computed tomography (CT) for stratifying patients with HCC to curative ablation or palliative treatment. Two reader groups (radiologists, R1 and R2) performed blind reads of CT and gadoxetic acid-enhanced MRI (contrast dynamics only). A truth panel, with access to clinical and imaging follow-up data, served as reference. Primary endpoint was non-inferiority (margin: 5% points) of MRI vs. CT (lower 95% confidence interval [CI] > 0.75) in a first step and superiority (complete 95% CI > 1) in a second step. The intent-to-treat population comprised 538 patients. Accuracy of treatment decisions was 73.4% and 70.8% for CT (R1 and R2, respectively) and 75.1% and 70.3% for gadoxetic acid-enhanced dynamic MRI. Non-inferiority but not superiority of gadoxetic acid-enhanced dynamic MRI versus CT was demonstrated (odds ratio 1.01; CI 0.97–1.05). Despite a theoretical disadvantage in wash-out depiction, gadoxetic acid-enhanced dynamic MRI is non-inferior to CT in accuracy of treatment decisions for curative ablation versus palliative strategies. This outcome was not subject to the use of additional MR standard sequences.

## 1. Introduction

Hepatocellular carcinoma (HCC) represents approximately 80–90% of all liver cancers [1]. In 2020, there were an estimated 52,450 new cases and 32,750 deaths from liver, intrahepatic bile duct, gallbladder, and other biliary cancer, combined, in the USA [2].

Guidelines on the recommended imaging techniques to diagnose HCC show geographical variations. For the radiological work-up of patients with suspected HCC, the Asia-Pacific guidelines recommend use of gadoxetic acid-enhanced hepatobiliary MRI (HBI) combined with dynamic imaging in the first line [3]. North American and European guidelines, by contrast, do not incorporate HBI features and focus exclusively on dynamic criteria: namely, contrast-medium enhancement in the arterial phase, with wash-out in the portovenous/venous phase [4,5]. The theoretical rationale for excluding HBI criteria in Western guidelines is that uptake of gadoxetic acid early in the venous phase (resulting in a so-called transitional phase), in the surrounding liver parenchyma, may lead to a wash-out characterization of lesions besides perfusion-related factors only and, therefore, decrease specificity [4,5]. This technical consideration has not been reproduced, so far, in a prospective trial incorporating a clinically meaningful study endpoint, such as treatment decision-making. 

The prospective, phase II, randomized, controlled SORAMIC study investigated sorafenib, in combination with microtherapy, in HCC patients (EudraCT 2009-012576-27, NCT01126645). A SORAMIC substudy compared gadoxetic acid-enhanced MRI against contrast-enhanced multislice CT for the accurate stratification of patients to local ablation (i.e., curative treatment) versus palliative treatment [6]. An analysis from the SORAMIC diagnostic cohort showed that gadoxetic acid-enhanced HBI MRI, including dynamic parameters as well as hepatobiliary phase (HBP), was superior to contrast-enhanced CT for treatment decision-making [7].

The purpose of the current analyses from the SORAMIC diagnostic cohort was to compare the accuracy of gadoxetic acid-enhanced dynamic MRI against contrast-enhanced CT when applying perfusion criteria only, i.e., following current Western guidelines. We sought to determine whether the theoretical limitation of obscured wash-out, employing gadoxetic acid for dynamic MRI, has a negative impact on treatment decisions in patients with HCC employing standard criteria with arterial wash-in and portovenous/venous wash-out. Eventually, this could support the clinical use of gadoxetic acid-enhanced contrast dynamics as part of multiparametric hepatobiliary liver MRI. 

## 2. Materials and Methods

SORAMIC is a prospective, phase II, open-label, multicentre, randomized trial conducted at 38 sites in 12 European countries. All procedures followed were in accordance with the ethical standards of the responsible committee on human experimentation (institutional and national) and with the Helsinki Declaration of 1975, as revised in 2008. Informed consent was obtained from all patients for being included in the study.

The SORAMIC diagnostic study had the primary objective to confirm, by a two-step procedure, that gadoxetic acid-enhanced HBP MRI is: (1) non-inferior or (2) superior to contrast-enhanced multislice CT for stratifying patients to palliative or local ablation strategies [7]. In this post-hoc analysis, we report the same endpoint, including identical biostatistical considerations, for the comparison of gadoxetic acid-enhanced dynamic MRI (i.e., a subset of the full HBI MRI) to contrast-enhanced CT, applying perfusion criteria only. Study methods for the SORAMIC diagnostic study are detailed in Ricke et al. [7].

Patients with confirmed HCC in Barcelona Clinic Liver Cancer (BCLC) stages A, B, and C, as well as Child–Pugh A through B7, were eligible for investigation by gadoxetic acid-enhanced MRI and contrast-enhanced CT. Allocation to the treatment strategy within the therapeutic study (curative versus palliative) was done independently at initial investigator assignment. The intent-to-treat (ITT) population included all patients undergoing both CT and MRI. The per-protocol population (PP) was defined by the absence of major image artefacts.

At baseline, patients underwent both CT and MRI within two weeks. At follow-up, patients assigned to the curative intent arm (independently at initial investigator assignment, see above) subsequently underwent MRI and CT every two months; patients in the palliative cohort did not follow a fixed imaging protocol. 

The CT protocol included pre-contrast, arterial, and portovenous phases of the upper abdomen, together with venous phase, of the whole abdomen. Contrast injection speed was 4 mL/s. The trigger delay for arterial and portovenous phases was 15 s and 50 s, respectively, after the bolus attained 100 HU in the descending aorta, while venous phase assessment was 120 s after contrast medium injection. The maximum accepted slice thickness was 5 mm.

The analysed MR protocol consisted of T1-weighted gradient echo sequences (T1-w GRE) and 3D (axial, slice thickness ≤ 5 mm) pre-contrast sequences. Following that, injection of gadoxetic acid was performed via rapid hand or power injector (1.5 mL/s) at a dose of 0.025 mmol/kg body weight, followed by a 30 mL saline flush. After injection, dynamic T1-w GRE 3D sequences were acquired in the late arterial phase (start of sequence via bolus tracking, intended start of central k-space readout 15 s after bolus detected in the descending aorta), portovenous phase (start 60–70 s after contrast injection), and venous phase (120 s after contrast injection); axial, slice thickness ≤ 5 mm (Supplementary Material File S1). In order to compare the diagnostic capacity of the dynamic criteria only, any additional MR sequence, such as T2 or diffusion-weighted imaging (DWI), was not the subject of analysis.

The CT and gadoxetic acid-enhanced dynamic MRI images were assessed in blinded fashion by two reader groups with >7 years of experience in abdominal diagnostic imaging: reader group 1 (R1), comprising 1 radiologist, and reader group 2 (R2), consisting of 6 radiologists. 

The truth panel consisted of a hepatologist and a radiologist at the same tertiary HCC and liver transplantation centre, both with >10 years of experience. The truth panel had access to baseline clinical data, as well as all CT and MRI images at baseline and during the first year after study inclusion. Decisions by the truth panel were made by consensus, with the inclusion of a second experienced radiologist in case no consensus was reached. 

Imaging criteria for HCC diagnosis included: lesion diameter > 1 cm with the presence of arterial enhancement and wash-out (‘typical HCC’) in the dynamic image data sets. The imaging criteria for performing a local ablation were: up to 4 lesions < 5 cm and an absence of macrovascular invasion (i.e., the criteria reported in the SORAMIC study [7]). Diagnostic confidence was assessed by a four-point scale.

Statistical analysis used SAS (version 9.4, SAS Institute, Cary, NY, USA) and the R-system for statistical computing (version 3. 5.1, R Foundation, Vienna, Austria). 

For determination of sample size, it was assumed that MRI and CT had accuracies of 80–85% and 80%, respectively. The non-inferiority margin of −5% points was equivalent to an odds ratio (OR) of 0.75. MRI was concluded to be non-inferior to CT, if the lower limit of the 95% confidence interval (CI) for the OR of the accuracies of MRI and CT was >0.75%, and MRI was considered to be superior to CT if the 95% CI was >1. The main efficacy variable was analysed with generalised estimated equations (GEEs) (SAS: proc genmod) and an independent working correlation matrix. A simulation study showed the non-inferiority endpoint had a power of 99.9% [7].

Quantitative variables were reported by descriptive statistics, and categorical data were expressed as absolute and relative frequencies. Forest plots depicted the ORs of accuracies in the data sets in association with confounding parameters. The Cis for ORs are two-sided and provide 95% confidence.

## 3. Results

**Figure 1:**
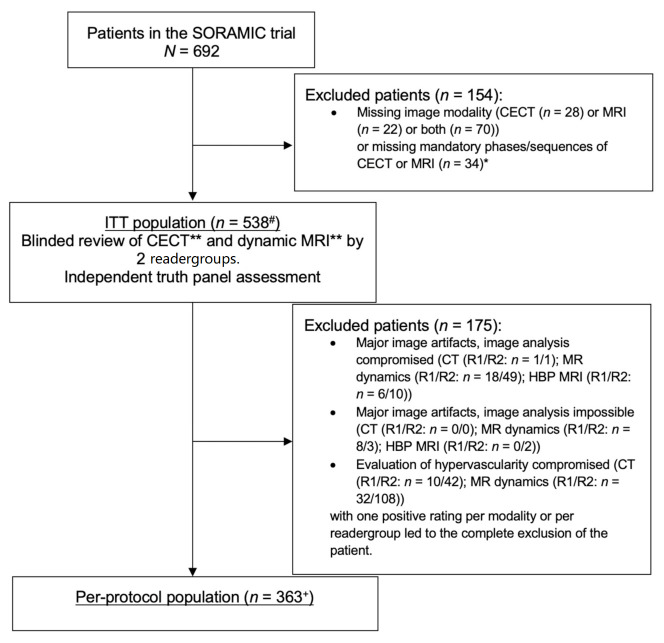
Flow diagram. * Mandatory phases/sequences were arterial, portovenous, and venous phases for contrast-enhanced CT (CECT) and axial T1 3D pre-contrast, arterial phase, portovenous phase, venous phase, and hepatobiliary phase (HBP), coronal T1 3D HBP, and axial T2 turbo spin echo (TSE) with or without fat saturation for MRI. ** Imaging criteria for HCC in CECT and MRI without HBP: wash-in and wash-out. ^#^ including 91 screening failures in the therapeutic study arms of the SORAMIC trial. ^+^ including 60 screening failures in the therapeutic study arms of the SORAMIC trial.

Patients were recruited into the SORAMIC diagnostic study between 5 January 2011 and 19 April 2016. The ITT and PP populations included 538 and 363 patients, respectively, for the diagnostic study, as well as for the post-hoc analysis described herein (Figure 1). The majority of patients were male (87%) and Caucasian (94%), with a median age of 66 years. Baseline characteristics are described in detail in Table 1. 

### 3.1. Accuracy of Treatment Decisions

The accuracy of treatment decisions in the ITT was 75.1% and 70.3% (R1 and R2) for gadoxetic acid-enhanced dynamic MRI and 73.4% and 70.8%, respectively, for CT (Table 2). The OR for gadoxetic acid-enhanced dynamic MRI versus dynamic CT was 1.01 (0.97–1.05), therefore demonstrating non-inferiority, but not superiority, between the techniques. In the PP population, the accuracies of treatment decisions were 79.1% and 72.2% for gadoxetic acid-enhanced dynamic MRI and 76.6% and 71.6% employing CT. Relevant imaging artefacts, therefore, had no impact on the accuracy of treatment decisions. Subgroup analysis of patients with histological verification of the disease (ITT population) again revealed no difference in the accuracy of treatment decisions between gadoxetic acid-enhanced MRI (78.5% and 74.0%, R1 and R2) and CT (76.2% and 75.3%); OR 0.99 (95% CI 0.94–1.05).

**Figure 2:**
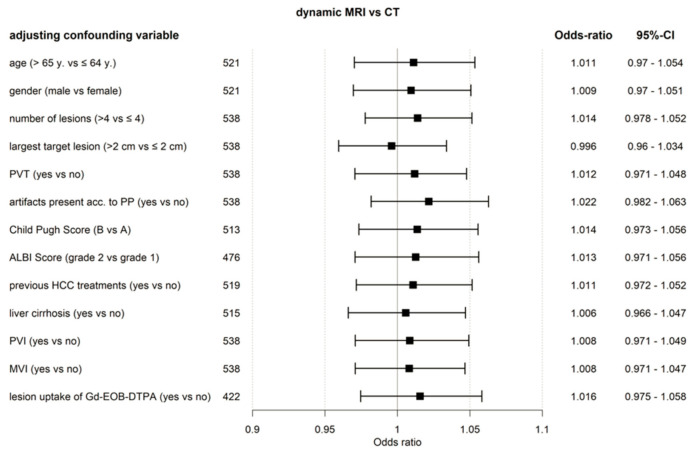
Forest plot. Accuracy of the treatment decision, dynamic MRI versus CT (ITT population) based on GEE model including confounding factors. ALBI, assessment of albumin-bilirubin; CT, computed tomography; Gd-EOB-DTPA, gadoxetic acid; HCC, hepatocellular carcinoma; MRI, magnetic resonance imaging; MVI, macrovascular invasion; PP, per protocol; PVI, portal vein infiltration; PVT, portal vein thrombosis; y, year.

Results of GEE analysis, including factors with a potential influence on the accuracy of treatment decisions, are shown in Figure 2. The non-inferiority of gadoxetic acid-enhanced dynamic MRI, compared to dynamic CT, was confirmed. 

Interreader agreement between the reader groups was moderate for CT (Cohen’s kappa: 0.58 [95% CI 0.51–0.66], R1 vs. R2, ITT) and substantial for dynamic MRI (0.67 [95% CI 0.61–0.74]). Results for the PP population were comparable between dynamic MRI and CT at 0.73 (95% CI 0.66–0.81) and 0.61 (95% CI 0.53–0.7), respectively.

### 3.2. Diagnostic Confidence 

Diagnostic confidence (i.e., combined percentages of ‘very confident’ and ‘confident’, ITT population) was inferior for gadoxetic acid-enhanced dynamic MRI, compared to CT for R1, and was similar for R2 (MRI: R1: 88.3%, R2: 86.9%; CT: R1: 95.7%, R2: 87.9%; OR R1: 0.3 [CI 0.2–0.6]; OR R2: 0.9 [CI 0.6–1.3]). 

### 3.3. Detection Rate of Lesions 

The dichotomized assessment of lesion number showed that CT identified 0–4 lesions in 65.8% (R1) and 62.6% (R2) of patients, compared to 67.7% (R1) and 66.5% (R2), respectively, by gadoxetic acid-enhanced dynamic MRI. Comparing gadoxetic acid-enhanced dynamic MRI and CT, the OR for correct assessment of lesion number 0–4 versus >4 was 0.9 (CI 0.7–1.1) for R1 and 0.8 (CI 0.7–1.1) for R2. Details on lesion detection rate and maximum lesion size are outlined in Table 3.

### 3.4. Artefacts 

Image quality of CT (good or average versus poor) was statistically superior, in both reader groups, to gadoxetic acid-enhanced dynamic MRI, with 99.8/99.4% (R1/R2) assessed as good in CT and 89.2/91.9% (R1/R2) in gadoxetic acid-enhanced dynamic MRI. Comparing the presence of relevant artefacts (major artefacts compromising the analysis or making analysis impossible versus no and minor artefacts) revealed statistically fewer cases for CT, compared to gadoxetic acid-enhanced dynamic MRI, in both reader groups (0.2/0.2% [R1/R2] vs. 4.9/9.7% [R1/R2]). CT showed significantly fewer cases of incorrect timing of the arterial phase compared to gadoxetic acid-enhanced dynamic MRI in both reader groups (3.3/14.9% [R1/R2] vs. 7.8/26.8% [R1/R2]). CT delivered significantly fewer cases in which the assessment of tumour hypervascularity was compromised (1.9/7.8% [R1/R2] vs. 5.9/20.1% [R1/R2]).

The PP population was defined by the absence of major artefacts compromising the analysis. In both reader groups, CT showed significantly fewer cases meeting this definition as compared to gadoxetic acid-enhanced dynamic MRI (1.9/8.0% [R1/R2] vs. 7.6/22.1% [R1/R2]). Details of the imaging artefacts are outlined in Table 4.

### 3.5. Portal Vein Thrombosis and Portal Vein or Other Macrovascular Invasion

Presence of portal vein thrombosis was identified in 34.4% (R1) and 41.5% (R2) of cases by CT and in 32.3% (R1) and 42.0% (R2) by gadoxetic acid-enhanced dynamic MRI (OR 0.9 [95% CI 0.7–1.2] for R1 and 1.0 [95% CI 0.8–1.3] for R2). Portal vein invasion/macrovascular invasion were identified in 31.6/32.7% (R1) and 30.9/35.3% (R2) by CT and in 29.4/30.5% (R1) and 30.5/35.5% (R2) by gadoxetic acid-enhanced dynamic MRI (OR 0.9 [95% CI 0.7–1.2] for R1 and 1.0 [95% CI 0.8–1.3] for R2).

## 4. Discussion

In this post-hoc analysis of the SORAMIC diagnostic study, we describe the non-inferiority of gadoxetic acid-enhanced dynamic MRI, compared to contrast-enhanced CT for the accuracy of treatment decision-making, in patients with HCC. HCC diagnosis was based on perfusion criteria only, as determined in current Western guidelines. All assessments, therefore, were based on dynamic MR and CT imaging (for arterial, portovenous, and venous phase only). A previously reported analysis from the SORAMIC diagnostic study concluded that hepatobiliary imaging (in addition to perfusion criteria) with gadoxetic acid-enhanced MRI was superior to CT in the accuracy of treatment decisions [7]. In addition to these former results, the post hoc analysis described herein confirms the non-inferiority of gadoxetic acid-based dynamic MRI versus contrast-enhanced CT for HCC perfusion imaging.

Current western guidelines use exclusively arterial and portovenous/venous criteria on contrast-enhanced MRI, with the aim to enhance the specificity of the MR technique [4,5]. Our study considered a signal decrease of a lesion, during the portovenous or venous phase (defined as 60–70 s and 120 s after gadoxetic acid injection, respectively), as positive for wash-out, when assessing the dynamic image data set of gadoxetic acid, and is in line with the recommendations in Western guidelines when applying extracellular MR contrast media [5,8]. However, the value of gadoxetic acid for HCC diagnosis based on perfusion criteria, and the criteria to be included, are under dispute, since early liver uptake of gadoxetic acid may obscure the depiction of true lesion wash-out, which led to a renaming of the venous phase to the transitional phase in gadoxetic acid liver MRI [4,9]. Retrospective studies have reported high sensitivity but a reduction in specificity when wash-out in the portovenous phase is combined with hypointensity in the transitional phase, measured 3 min after injection (decrease in sensitivity: from 97.9% to 86.3% [10], 92.9% to 78.6% [11], and 100% to 94.9% [12], while two other retrospective studies have interpreted that extending the wash-out appearance to transitional phase or HBP, rather than portovenous phase alone, allows high sensitivity without significant reduction in specificity (decrease in specificity: from 94.1% to 82.0% [13] and 90.9% to 84.8% [14]), in support of our findings. 

Numerous studies have compared gadoxetic acid-enhanced HBI MRI versus contrast-enhanced CT in HCC, with the conclusion that the former technique consistently provides superior lesion detection [13,15,16,17,18]. There is, however, a paucity of data that directly compare gadoxetic acid-enhanced dynamic MRI against CT, without addition of further MR sequences such as T2 and DWI. A recent study by Semaan et al. reported per-lesion sensitivities for HCC detection of 59.5% for CT versus 69.7% for gadoxetic acid-enhanced dynamic MRI, which were both lower than 76.8% for gadoxetic acid-enhanced MRI, including HBI [19]. To the best of our knowledge, our study is the first to relate dynamic contrast-enhanced MRI to a clinical decision endpoint. 

Uptake of gadoxetic acid in the late phase is seen in approximately 10% of HCC lesions [20]. Theoretically, this uptake, potentially already starting in the transitional phase, can pose a bias in the evaluation of venous wash-out in gadoxetic acid-enhanced liver MRI. However, lesion uptake of gadoxetic acid in the late phase was not a significant confounding factor in our analysis (Figure 2).

Gadoxetic acid-enhanced dynamic MRI appeared to perform independently of a number of influencing factors (Figure 2). Compromised imaging in the arterial phase was reported in up to 20.1% patients (reader group 2) in the gadoxetic acid group (a sum of incorrect timing and movement artefacts), in the majority of cases most probably related to transient severe motion (TSM). Similar or lower rates of TSM have been reported in the literature [21,22]. Although patients showed significantly more imaging artefacts with gadoxetic acid-enhanced MRI than CT, treatment decision-making was not impaired in both the ITT and PP populations. 

Trial limitations include the lack of histopathology to confirm the truth panel assessment in approximately one-half of patients. We consider, however, that potential bias should not be a concern, in view of the large data sets available before and after treatment. Secondly, more advanced tumour stages were more frequent than earlier tumour stages in our trial. However, a higher rate of advanced tumour stages reflects real life in HCC diagnosis [5]. Thirdly, the inclusion criteria in SORAMIC do not reflect the current guidelines for local ablation. However, we propose that these expanded criteria for ablation, regarding size and number of lesions, do not affect the study outcomes, which result from appropriate lesion identification. Our study does not include the LI-RADS classification. LI-RADS was adopted in the AASLD guidelines in 2018 [8], and further validation is ongoing, most likely leading to a powerful tool for standardized HCC diagnosis. However, at protocol development and Statistical Analysis Plan generation, LI-RADS had not yet been established. LI-RADS, therefore, was not included in our blind read. We suggest LI-RADS would not add benefit to our analysis since it comprises perfusion criteria only. T2 sequences, as well as DWI today, are part of the recommended MR protocols [4,8]. These sequences were not part of the study hypothesis. Inclusion of T2 sequences and DWI would likely have improved the performance of MR in our study.

## 5. Conclusions

Gadoxetic acid-enhanced dynamic MRI (employing arterial, portovenous, and venous phase) was shown to be non-inferior to CT in the accuracy of treatment decisions for curative ablation versus palliative strategies. Information on gadoxetic acid-enhanced MRI contrast dynamics may, therefore, be used for HCC diagnosis, based on perfusion criteria, supporting the use of gadoxetic acid-enhanced multiparametric liver MRI in the diagnosis and surveillance of HCC.

## Figures and Tables

**Table 1:** Demographics and baseline characteristics.

Parameter		Median	IQR	*n*	Valid%
Sex (17 ^a^)	Women			69	13.2
	Men			452	86.8
Age (y) (17 ^a^)		66	59–73		
	≤65			249	47.8
	>65			272	52.2
Race (38 ^a^)	Caucasian			468	93.6
	Other			32	6.4
Previous HCC treatment (19 ^a^)	Yes			150	28.9
	No			369	71.1
Previous HCC treatments in detail	TACE or TAE			102	19.7
	Resection			44	8.5
	PVE, no resection			4	0.8
	Local ablation			51	9.8
Liver cirrhosis (23 ^a^)	Yes			418	81.2
	No			97	18.8
ECOG (31 ^a^)	0			375	74
	1			123	24.3
	≥2			9	1.8
HCC diagnosis by (19 ^a^)	Histology			223	43
	Imaging criteria			291	56.1
	Other			5	0.9
Cause of disease ^b^	Alcohol abuse			225	41.8
	Hepatitis B			57	10.6
	Hepatitis C			128	23.8
	NASH			49	9.1
	NAFLD			27	5
	Hemochromatosis			15	2.8
	Cryptogenic			50	9.3
	Other			6	1.1
	Alcohol abuse only			182	33.8
	Hepatitis B or C only			149	27.7
	No hepatitis B or C, no alcohol abuse			125	23.2
	Hepatitis B or C and alcohol abuse			25	4.6
Child-Pugh points (24 ^a^)	5 (A)			330	64.2
	6 (A)			127	24.7
	7 (B)			47	9.1
	8 (B)			6	1.2
	10 I			2	0.4
BCLC stage (25 ^a^)	0			6	1.2
	A			93	18.1
	B			144	28.1
	C			269	52.4
	D			1	0.2
Metastases (21 ^a^)	y			90	17.4
	*n*			427	82.6
Specified	Lymph node			49	9.5
	Bone			10	1.9
	Other			31	6
Study arm ^c^	Curative arm			95	17.7
	Palliative arm			354	65.8
	Screen failure			89	16.5
No. of patients by country (No. of centres)	Germany (10)			226	42.0
	Switzerland (1)			3	0.6
	Austria (2)			25	4.7
	The Netherlands (1)			54	10
	Poland (3)			32	5.9
	Belgium (1)			10	1.9
	Spain (1)			28	5.2
	Turkey (1)			10	1.9
	Great Britain (4)			24	4.5
	France (5)			70	13.0
	Italy (3)			40	7.4
	Slovenia (1)			16	3.0

^a^ Number of missing cases; reflect screening failures of the therapeutic study part of the SORAMIC study, ^b^ multiple answers possible, ^c^ by decision of the local investigators. BCLC, Barcelona Clinic Liver Cancer; ECOG, Eastern Cooperative Oncology Group; HCC, hepatocellular carcinoma; IQR, interquartile range; *n*, number; NAFLD, non-alcoholic fatty liver disease; NASH, non-alcoholic steatohepatitis; PVE, portal vein embolization; TACE, transarterial chemoembolization; TAE, transarterial embolization; y, years.

**Table 2:** Accuracy of treatment decision and comparison of modalities.

**(a) Accuracy of Treatment Recommendation ^a^**				
	**CT**		**Gadoxetic acid-enhanced dynamic MRI**	
	**Reader 1**	**Reader 2**	**Reader 1**	**Reader 2**
Accuracy of treatment recommendation ITT (*n* = 538) ^a^	73.4%	70.8%	75.1%	70.3%
Accuracy of treatment recommendation per protocol (*n* = 363) ^a^	76.6%	71.6%	79.1%	72.2%
Accuracy of treatment recommendation histological verified cases only (*n* = 223) ^a^	78.5%	74.0%	76.2%	75.3%
**(b) OR by Modality and Reader Group**				
	**CT**			
	**Reader group 1**		**Reader group 2**	
	**OR**	**CI (LCI-UCI)**	**OR**	**CI (LCI-UCI)**
ITT				
Gadoxetic acid-enhanced dynamic MRI as compared to	1.09	0.83–1.43	0.97	0.75–1.27
Per Protocol				
Gadoxetic acid-enhanced dynamic MRI as compared to	1.15	0.81–1.64	1.03	0.74–1.42
ITT histological verified cases only				
Gadoxetic acid-enhanced dynamic MRI as compared to	0.88	0.56–1.37	1.07	0.70–1.65
**(c) OR by Modality (Based on GEE with Independent Working Correlation Matrix)**				
	**CT**			
	**OR**		**CI (LCI-UCI)**	
ITT				
Gadoxetic acid-enhanced dynamic MRI as compared to	1.01		0.97–1.05	
Per Protocol				
Gadoxetic acid-enhanced dynamic MRI as compared to	1.02		0.98–1.07	
ITT histological verified cases only				
Gadoxetic acid-enhanced dynamic MRI as compared to	0.99		0.94–1.05	

^a^ As compared to truth panel. CI, confidence interval; CT, computed tomography; GEE, generalized estimating equation; ITT, intent to treat; LCI, lower confidence interval; MRI, magnetic resonance imaging; OR, odds ratio; UCI, upper confidence interval.

**Table 3:** Lesion characteristics.

Lesion Detection CT and MRI Imaging				
	CT		Gadoxetic Acid-Enhanced Dynamic MRI	
	R1 (*n*/%)	R2 (*n*/%)	R1 (*n*/%)	R2 (*n*/%)
**Patients with lesions > 1 cm with arterial enhancement/wash-out (*n*/%)**				
Lesion number *n* = 1	194/36.1	181/33.6	202/37.6	173/32.2
Lesion number *n* = 2–4	138/25.7	118/21.9	128/23.8	145/27.0
Lesion number *n* = 5–20	113/21.0	140/26.0	112/20.8	135/25.1
Lesion number *n* > 20	71/13.2	61/11.4	55/10.2	45/8.4
Longest hypervascularized diameter lesions (cm, mean/SD)	6.1/4.1	5.1/3.6	7.0/4.4	5.4/3.7
Rate of lesion number 0–4	354/65.8	337/62.6	363/67.7	358/66.5
Rate of lesion number > 4	184/34.2	201/37.4	174/32.3	180/33.5

CT, computed tomography; MRI, magnetic resonance imaging.

**Table 4:** Imaging artefacts.

**Image Quality and Artefacts, Frequencies**					
		**CT**		**Gadoxetic Acid-Enhanced Dynamic MRI**	
		***n***	**Valid %**	***n***	**Valid%**
Image quality (R1/R2)	Good or average	537/535	99.8/99.4	480/489	89.2/91.9
	poor	1/3	0.2/0.6	58/49	10.8/9.1
Artefacts present ^a^ (R1/R2)	Yes	1/1	0.2/0.2	26/52	4.9/9.7
Correct timing contrast dynamics (R1/R2)	Yes	520/458	96.7/85.1	496/394	92.2/73.2
Evaluation of hypervascularity compromised (R1/R2)	Yes	10/42	1.9/7.8	32/108	5.9/20.1
Combined artefacts (according to per protocol) ^b^ (R1/R2)	Yes	10/43	1.9/8.0	41/119	7.6/22.1
**Image quality and artefacts, comparison of modalities**					
		**CT**			
		**Reader 1**		**Reader 2**	
		**OR**	**CI (LCI/UCI)**	**OR**	**CI (LCI/UCI)**
Image quality good/average vs. poor	Gadoxetic acid-enhanced dynamic MRI as compared to	0.1	0.1/0.1	0.1	0.1/0.1
Artefacts present ^a^, yes vs. no	Gadoxetic acid-enhanced dynamic MRI as compared to	27.3	3.7/201.7	57.5	7.9/417.2
Correct timing contrast dynamics, yes vs. no	Gadoxetic acid-enhanced dynamic MRI as compared to	0.4	0.2/0.8	0.5	0.4/0.7
Evaluation of hypervascularity compromised, yes vs. no	Gadoxetic acid-enhanced dynamic MRI as compared to	0.3	0.2/0.6	0.3	0.2/0.5
Combined artefacts (according to per protocol) ^b^, yes vs. no	Gadoxetic acid-enhanced dynamic MRI as compared to	4.4	2.2/8.8	3.3	2.3/4.7

^a^ Affecting the image analysis (major AF, image analysis compromised or impossible), ^b^ major AF, image analysis compromised or impossible and/or evaluation of hypervascularity compromised. AF, artefact; CT, computed tomography; MRI, magnetic resonance; R, reader group.

## Data Availability

Data are available from the corresponding author on reasonable request.

## Supplementary Materials

The following supporting information can be downloaded at: https://www.mdpi.com/article/10.3390/curroncol29020051/s1, Supplementary File S1: Gadoxetic acid MRI protocol.
