# Supplementary material for: Gadoxetic Acid-Based MRI for Decision-Making in Hepatocellular Carcinoma Employing Perfusion Criteria Only—A Post Hoc Analysis from the SORAMIC Trial Diagnostic Cohort
Source: Curr Oncol. 2022 Jan 27;29(2):565–77. doi: 10.3390/curroncol29020051 (PMC8870455; doi:10.3390/curroncol29020051)
Supplement: Supplementary file 1 [file curroncol-29-00051-s001.zip › curroncol-1410304-supplementary.pdf]

Supplementary File S1: Gadoxetic acid MRI protocol

The gadoxetic acid-enhanced MRI protocol consisted of T1-weighted gradient echo sequences (T1-w GRE), 2D (axial, slice thickness ≤6 mm) and T1-w GRE, and 3D (axial, slice thickness ≤5 mm) pre-contrast sequences. Injection of gadoxetic acid was performed via rapid hand or power injector (1.5 mL/s) at a dose of 0.025 mmol/kg body weight followed by a 30 mL saline flush. After injection, dynamic T1-w GRE 3D sequences were acquired in the late arterial phase (start of sequence via bolus tracking, start of central k-space readout 15s after bolus detected in the descending aorta), portovenous phase (start 60–70s after contrast injection), and venous phase (120s after contrast injection); all axial, slice thickness ≤ 5mm. These acquisitions were followed by a T2-weighted turbo spin-echo (T2-w TSE), 2D sequence with and without fat suppression (axial, slice thickness ≤8 mm). DWI was added with recommended b-values of 0, 100, 600, and 1000 s/mm^2. In the HBP at 20 min post-contrast injection, two T1-w GRE 3D sequences were performed (coronal, slice thickness ≤6 mm, and axial, slice thickness ≤5 mm), followed by a T1-w GRE 2D (axial, slice thickness ≤6 mm). Independent reading included the following mandatory sequences: axial T1-w GRE 3D pre-contrast, arterial, portovenous, venous, and hepatobiliary phases; coronal T1w GRE 3D, hepatobiliary phase; and axial T2-w TSE, with/without fat saturation.
